# Faculty drivers and barriers: laying the groundwork for undergraduate STEM education reform in academic departments

**DOI:** 10.1186/s40594-017-0062-7

**Published:** 2017-04-13

**Authors:** Susan E. Shadle, Anthony Marker, Brittnee Earl

**Affiliations:** Boise State University, 1910 University Dr., Boise, ID 83725 USA

**Keywords:** Barriers to change, Drivers to change, STEM education reform, Evidence-based instructional practices, Departmental differences

## Abstract

**Background:**

Calls to improve student learning and increase the number of science, technology, engineering, and math (STEM) college and university graduates assert the need for widespread adoption of evidence-based instructional practices in undergraduate STEM courses. For successful reforms to take hold and endure, it is likely that a significant shift in culture around teaching is needed. This study seeks to describe the initial response of faculty to an effort to shift teaching norms, with a long-term goal of altering the culture around teaching and learning in STEM. While the effort was envisioned and led at the institutional level, dialog about the proposed change and actions taken by faculty was emergent and supported within departments.

**Results:**

Faculty identify a variety of barriers to proposed changes in teaching practice; however, faculty also identify a variety of drivers that might help the institution alter teaching and learning norms. Analysis of faculty responses reveals 18 categories of barriers and 15 categories of drivers in faculty responses. Many of the barrier and driver categories were present in each department’s responses; however, the distribution and frequency with which they appear reveals departmental differences that are important for moving forward with strategies to change teaching practice.

**Conclusions:**

Addressing faculty’s barriers to change is essential, but identifying and leveraging faculty’s drivers for the change is potentially equally important in efforts to catalyze changes that are supported or constrained by the local context. Further, the collection of faculty perspectives opens a dialog around the current and future state of teaching, an important step in laying the groundwork for change. Departmental differences in barriers and drivers make clear the importance of “knowing” the local contexts so strategies adopted by departments can be appropriately tailored. Results are discussed in light of what kind of strategies might be employed to effect changes in STEM education.

**Electronic supplementary material:**

The online version of this article (doi:10.1186/s40594-017-0062-7) contains supplementary material, which is available to authorized users.

## Background

A significant body of research has focused on understanding the challenges associated with the propagation of evidence-based instructional practices (EBIPs) among science, technology, engineering, and math (STEM) faculty in higher education. The majority of this research has focused on the variety of contextual factors that present barriers to changing an individual’s teaching practices (Henderson and Dancy 2007). For example, a perceived lack of weight placed on teaching effectiveness in personnel decisions, lack of nontraditional assessments of teaching effectiveness, and a lack of pedagogical training present barriers to instructional innovation (Walczyk et al. 2007). Also, student expectations or resistance, lack of time, and concerns about covering the course content are noted in the literature (Henderson and Dancy 2007; Parker et al. 2015; Brownell and Tanner 2012; Andrews and Lemons 2015). Importantly, individual and contextual factors may be discipline- or department-dependent, suggesting generalization of barriers, or a one-size-fits-all approach to change, may not be appropriate (Lund and Stains 2015).

There has been less work to establish what might drive faculty to adopt EBIPs. Many factors that have been identified as drivers are inferred based on being the opposite of a barrier. For example, if the current faculty reward system is a barrier, then it can be asserted that changing the reward system would help drive change. However, Hertzberg (2003) postulated that the opposite of dissatisfaction is not satisfaction and vice versa; rather, barriers and drivers are separate factors that need to be accounted for individually. For instance, lack of time may be a barrier to adopting EBIPs, but the availability of more time will not necessarily drive greater adoption of EBIPs; it merely enables it. There are, however, some recent studies that have looked more proactively at drivers. Andrews and Lemons (2015) determined that self-considerations (personal satisfaction and self-image), interactions with other people (students and colleagues), and contextual factors (e.g., the need for teaching materials) were the primary drivers for the adoption of a particular pedagogical approach. Dissatisfaction has also been identified as a critical element in driving successful pedagogical changes; unless one is dissatisfied with one’s current pedagogy, there is little reason to change current practices (Gess-Newsome et al. 2003). Lund and Stains (2015) also documented supportive influences to the adoption of EBIPs, such as prior pedagogical experiences and faculty’s personal teaching attitudes and beliefs. However, whether these were actually supportive influences rather than impeding influences was discipline- or department-dependent.

Despite our relatively thorough understanding of the barriers, as well as insights that have emerged from studies that have looked at drivers, reliable strategies that can be implemented to catalyze the widespread adoption of EBIPs in higher education have yet to be identified (Wieman and Gilley 2013; Hastings and Breslow 2015; National Science Foundation 2013). For successful reforms to take hold, it has been suggested that a significant cultural shift around teaching is needed (Gess-Newsome et al. 2003; Brownell and Tanner 2012). This indicates that change will be a complex process and will require strategies focused on something broader than the adoption of evidence-based pedagogies in the classroom. While research indicates that the decision to make changes to one’s teaching occurs at an individual level (Gess-Newsome et al. 2003; Andrews and Lemons 2015; Dormant 2011; Bouwma-Gearhart 2012), the larger context(s) in which faculty make decisions about teaching are important. It is likely that efforts to implement strategies at both the institutional and department level will be needed. Henderson et al. (2011) found the most common change strategies documented in STEM education reform efforts fall in to one of the following categories: dissemination of curriculum and pedagogy, encouragement of teachers’ use of their own expertise to improve instruction, and the enactment of new policy. A fourth category, which was observed to be utilized less frequently, focuses on creating “a collective vision… that will support new modes of instruction.” This fourth category contains strategies which are targeted at the institutional contexts and for which the outcome of the strategy is emergent (Henderson et al. 2011). Such strategies are aimed at involving faculty (and others) in negotiating a process by which new normative assumptions and practice around teaching can emerge. It highlights the important role of engaging a group of individuals that is both diverse and on “the front lines” (Borrego and Henderson 2014). The assertion is that people will be more likely to adopt changes in which they are able to participate; these are changes done “with” them, compared to changes that are dictated from higher up the organizational chart or changes done “to” them (Oreg et al. 2011; Dormant 2011). Despite its potential, the applicability of strategies for change in the shared vision category is not well understood but may have the greatest potential to be transformative (Borrego and Henderson 2014).

This study seeks to describe the initial response of faculty to an effort to shift teaching norms in STEM, with a longer-term goal to alter the culture around teaching and learning in STEM. While the effort was envisioned and led at the institutional level, dialog about the proposed change and actions taken by faculty emerged from dialog within the departments. The purpose of the activity described here was to open a dialog with faculty to better understand faculty responses to recommendations for STEM education reform that have emerged at the national level.

The project began with a group of campus leaders brainstorming a set of behaviors we would expect to observe if STEM teaching norms were to shift from teacher centered to student centered (Kember 1997; Trigwell and Prosser 2004; Weimer 2002). Certainly, some faculty were already using student-centered approaches, but at the time of this study, teacher-centered approaches were decidedly the norm (Stieha et al. 2016). To move toward the vision, faculty conceptions about teaching and learning, the assumptions they make around how teaching and learning works, and what teaching looks like may need to change (Czajka and McConnell 2016; Kember 1997). The leadership group understood that in order to move toward this vision, faculty would need an opportunity to “buy-in” and to operationalize the vision for themselves individually and within their departmental context (Kezar 2013). The vision (*vide infra*) intentionally focuses on a collection of behaviors because it was intended to provide broad but concrete ideas of what a “future state” could look like. The choice to go beyond the adoption of EBIPs was also intended to capture the broad nature of the shift in norms that was envisioned by the leadership team.

The specific questions this study seeks to address are the following: how do faculty respond to a proposed shift in normative teaching and learning practices? What ideas do faculty express that represent barriers to the vision? What ideas serve as drivers toward the vision? How does the faculty response vary across departments? The results are discussed in light of implications for stimulating change in STEM education.

## Methods

### Data collection

The data collection performed for this study was part of a large NSF-funded STEM education reform project. Data were initially collected during the Spring 2014 semester in department meetings in the following ten STEM departments in two different colleges at Boise State University: Biological Sciences, Chemistry, Civil Engineering, Computer Science, Electrical and Computer Engineering, Geosciences, Materials Science and Engineering, Mathematics, Mechanical and Biomedical Engineering, and Physics. Data was collected later during Fall 2015 for two additional departments, which had recently been moved into the College of Arts and Sciences: Anthropology and Psychology. Institutional Review Board approval was secured for this study. The reason for conducting these meetings at the department level was both to engage as many faculty in the discussion as possible and to identify differences in department responses.

The framework for collection of data and the prompts used were based on “The Chocolate Model of Change” (Dormant 2011) which stresses the importance of partnering with and collecting information from adopters of a proposed change. The protocol used was piloted with project leadership and with the project advisory board before inviting faculty participation. Each meeting began with an introduction of the institutional STEM education reform project and its vision statement (below). Faculty were also informed that the purpose of the meeting was to collect their responses to the vision. Further, they were told that as the project unfolded, departments would be supported to engage in departmentally driven, local projects and activities to help move toward the vision. At this stage of the process, no other specific action items or program details were introduced. The overall effort engaged faculty in thinking about the vision as a possible destination and provided an invitation for faculty to consider their participation, which would be voluntary (Marker et al. 2015). It is important to note that the vision statement was not introduced as a “top-down” mandate.

**Table:** 

VISION STATEMENT: The culture of teaching and learning at Boise State will be characterized by • on-going exploration and adoption of evidence-based instructional practices • faculty engaged in continuous improvement of teaching and learning • dialogue around teaching supported through a community of practice • teaching evidenced and informed by meaningful assessment The fulfillment of this vision will enhance our learning-centered culture and will result in increased student achievement of learning outcomes, retention, and degree attainment; especially among underrepresented populations	

The primary activity of the meeting was to ask participants to read the vision statement and consider movement toward this “end state.” In particular, in accordance with Dormant’s (2011) change framework, participants were asked to consider five characteristics of the proposed change (e.g., movement toward the vision): its relative advantage, simplicity, compatibility, flexibility, and social impact. The facilitators intentionally did not take time to build a shared understanding of the vision in order to allow ideas to surface that would illuminate faculty’s interpretation of the vision and identify their perceived barriers and drivers. Participants were provided a handout which contained several prompts related to the five change characteristics (see Table 1). Information about each characteristic was collected as follows: after a brief description of the characteristic and examples of both positive and negative responses, participants were asked to individually write down ideas in response to each prompt (see Table 1). Example responses can be found in Tables 2 and 3. After individual responses were generated, volunteers were asked to share responses they felt were most important and a short facilitated discussion ensued. The purpose of the discussion was to provide an opportunity for faculty to share ideas. This dialog was intentionally envisioned as part of the change process itself; in the discussion, faculty illuminated ideas that alluded to both current and envisioned teaching norms. After the discussion, individuals had an opportunity to add additional comments to their response sheet.

**Table 1:** Change protocol meeting prompts

Change characteristic (Dormant 2011)	Prompt
Relative advantage	1a. Ways in which this end state is advantageous to me/my department
	1b. Ways in which this end state is disadvantageous to me/my department
Simplicity	2a. Features of our current environment and practice that make this end state easy/simple to attain and/or maintain
	2b. Features of our current environment and practice that make this end state/hard complex to attain and/or maintain
Compatibility	3a. Ways in which the end state is compatible with what I already do
	3b. Ways in which the end state is incompatible with what I already do
Flexibility	4a. In what ways might the end state allow for flexibility and individual choice (while still achieving the vision)?
	4b. In what ways might the end state limit flexibility and individual choice in order to achieve the vision?
Social impact	5a. How will the new end state positively impact my relationships (with colleagues, with students, with administrators, etc.)?
	5b. How will the new end state negatively impact my relationship (with colleagues, with students, with administrators, etc.)?

**Table 2:** Categories of faculty-identified barriers for STEM education change

Barrier category	Description of category	Example faculty comments
Time constraints	Faculty is currently over-committed and does not have time to take on any more initiatives; working capacity is limited and involvement must be prioritized given other commitments	1) The amount of time available to “think about teaching” in a department where almost all of us are teaching in overload situations is not currently tenable; 2) There is limited time, so as more time is spent developing teaching materials less time is spent in other activities critical to one’s success as a faculty member
Instructional challenges	Inability to cover necessary content if EBIPs are used, inability to manage EBIPs and assessment in large enrollment courses, classroom space is not conducive to EBIPs due to fixed furniture or layout	1) Covering essential content in the face of decreased number of credits in the curriculum; 2) Course size limits many teaching practices (meaningful assessment in a class of 278 that does not swallow me whole)
Loss of autonomy	Perceived loss of autonomy in the classroom or over content; concern that one will be forced to use “one-size-fits-all” approaches with an increasing top-down management style	1) Force faculty to teach and assess all the same way, may not be best for their style; 2) Less individual control of content and methods
Resistance to change	No reason to change current practices; currently engaged in other changes (do not want to change more things); is resistant to change in general	1) I already get high teaching reviews, for purposes of the university promotional process; 2) I don't want to have to change my teaching style
Insufficient assessment methods and processes	Concern about how the administration will assess teaching effectiveness; concern about how faculty will assess learning in their classroom and/or determine if EBIPs result in improved student learning	1) Developing knowledge of meaningful assessment; 2) Emphasis on student evaluations as single measure
Inadequate resources	Lack of resources needed to explore and adopt EBIPs (e.g., teaching assistants to help in the classroom or with grading, materials, adequate learning spaces)	1) Resource requirements for change deplete limited pool; 2) Change needed in resources - infrastructure
Conflicts with institutional rewards/priorities	The tenure and promotion criteria are misaligned with the proposed initiative, research output carries more weight than teaching-related duties, and/or there is little incentive to focus more effort on teaching	1) Not so beneficial to me personally, in that teaching is not in my experience a strong criterion for obtaining tenure and promotions; 2) There is no reward for investing more in teaching
Student resistance	Students resist EBIPs; this might impact end-of-course evaluations	1) A population of students will be resistant to change; 2) Students don’t always evaluate change or “new” things in a positive or constructive way (and evaluations impact promotion and tenure)
Current culture is unsupportive	Department, institution, or higher ed. culture does not support pedagogical exploration, deviations from traditional lecture, and/or communities of practice	1) No current culture of experimentation; 2) We don't currently discuss as a department teaching practices
Competes with research	Potential adopters’ priorities lie in research and the proposed initiative compromises their ability to devote their time to research	1) Movement towards teaching changes culture & not necessarily positive (research needs to maintain its level of respect); 2) It will take valuable time to implement. This is time spent away from research used to judge my work
Departmental divisions	Concern that initiative will create departmental divides and negatively impact the social structure	1) Colleagues will evaluate each other's teaching, leading to conflict; 2) Will this change the tone of the faculty position
Lack of pedagogical skills/information	There is a lack of knowledge about EBIPs; knowledge and skills are needed to identify and implement appropriate EBIPs	1) Time necessary to keep up with EBIP research; 2) Understanding & having time to research correct tool
Lack of confidence in EBIPs	Validity of research or claims that support the use of EBIPs is in question	1) Doubts about outcomes/effectiveness; 2) Evidence based instructional practices are a fallacy
Underprepared students	Students lack the knowledge, skills, and/or motivation to be able to successfully engage in EBIPs	1) Seems that students are more concerned about exam grades then understanding the material; 2) Students are hard-wired to standard learning environments
Rigid or ambiguous nature of EBIPs	Lack of agreement about the appropriateness of various EBIPs	1) Formalized use of teaching tool for the incorrect application; 2) Conflict between faculty- lack of agreement on methods/standards
Vague end state/process to get there	Indicates initiative and proposed end state lacks clarity	1) Uncertainty of goals (on retention); 2) Vague goals, why not concrete quantitative objectives
Challenges in engagement across faculty rank	Departments may find it difficult to implement the initiative with faculty and teaching assistants not on the tenure track	1) Grads teach many labs w/o link to faculty; 2) No/little dialogue for adjuncts
Misalignment with accreditation requirements	Proposed initiative is misaligned with accreditation requirements and/or may interfere with accreditation efforts	1) Required to complete Accreditation Board for Engineering and Technology (ABET) … results w/the course; 2) Curriculum dictated (somewhat) by American Chemical Society (ACS)

**Table 3:** Categories of faculty-identified drivers for STEM education change

Driver category	Description of category	Example comments
Expands on current practices	Faculty member or other colleague(s) have already adopted EBIPs and/or are engaged in assessment to improve teaching	1) We already think about a lot of this stuff due to accreditation and dept. college culture; 2) Some faculty are already trying new techniques
Encourages collaboration and shared objectives	Collaboration and communities of practice is a beneficial outcome of increased emphasis on teaching and student success; development of shared vision	1) Agree on higher academic standards; 2) Some faculty could work together on course development and improvement
Improves teaching and assessment	Expectation for gains in individual teaching ability, confidence, and/or efficiency; more consistent curriculum across sections/department; better assessment processes	1) Improvements in instruction across the whole dept.; 2) Consistency in expectations of learning
Aligns with existing resources	Resources and materials are readily available to assist in the adoption/implementation of EBIPs: people, CTL, technology	1) Lots of support from the Center for Teaching and Learning and department; 2) Adoption of blackboard/video capture make evidence-based learning more feasible
Provides flexibility and encourages exploration	Adoption of new teaching practices fosters creativity; exploration/innovation are encouraged	1) Leaves room for personal innovation & experimentation; 2) Can explore effectiveness and compatibility w/best practices, with your teaching style & personality
Improves student and department outcomes	Realization of vision will result in improved outcomes for students and/or the department (e.g., student retention, decreased failure rates, fewer repeating students)	1) Will help improve student retention/graduation rates; 2) Successful results (That students performance or satisfaction improves)
Promotes student engagement and faculty-student interactions	There will be improved relationships/rapport with students; students enjoy active learning environments and will be more engaged	1) As teaching improves, relationships with students probably also improve; 2) With students: increase dialogue in classroom
Aligns with faculty desire for student success	Instructors are willing to try new things and have a shared desire for student success; aligned with current efforts for teaching effectiveness and improved student learning	1) Intrinsic motivation to prepare future citizens; 2) We/I'm motivated to push for better learning/retention
Develops stronger students/graduates	The use of active learning pedagogies will aid students in the development of skills necessary for future course work and employment	1) Relevance for students (skills needed outside Higher education); 2) Success of higher education in preparing thinkers and leaders
Institutional/departmental support	Vision is valued and supported by the department and/or institution; teaching will be valued in tenure and promotion process	1) Support from management Chair/Dean in testing new ideas; 2) Teaching quality is considered in T & P decisions
Encourages professional development	The proposed initiative is an opportunity to engage in professional development related to teaching and learning	1) Emphasis on training in teaching for faculty & grad students; 2) Faculty are supported to attend workshops even outside the university
Enhances teaching satisfaction	Faculty will experience greater satisfaction in their teaching roles	1) Enthusiasm - more energy in department; 2) More fun/fulfilling for faculty members
Improved individual and institutional reputation	Better teaching and improved student success will elicit greater recognition for the institution or individuals	1) Improving teaching improves recruitment and department reputation among students in particular; 2) Potentially provides better overall regional and national recognition
Builds common tools and resources	The creation/availability of common tools and resources is a valuable outcome of the proposed initiative	1) Successful strategies will be available to all; 2) Provides a “toolbox” for achieving learning
Increased research opportunities	The vision will expand research and/or is a means to connect teaching with research	1) I will explore additional topics that would help my research; 2) Could lead to collaboration on grants

The process then moved on to the next characteristic, and the steps (including discussion) were repeated. Each participant’s handout was collected at the end of the meeting. The Fall 2015 meetings were modified slightly based on our experience with the analysis of the Spring 2014 data. In these meetings, participants used a slightly modified version of Nominal Group Technique (Dunham 1998; McMillan et al. 2016). In it, they were introduced to the project and its vision as before. Then, the facilitator discussed all five characteristics and examples to frame the types of responses that might be elicited by different characteristics. Participants were then asked to write down their responses to each characteristic (individually). After individual responses were generated, participants were asked to share their recorded responses until all the unique views were reflected in two aggregate lists—one focused on barriers and one on drivers. The aggregated lists were recorded on chart paper on the wall. Participants then “voted” for the three most important ideas by putting a checkmark next to the three items they felt were most important to them. This produced a prioritized, aggregate list of ideas from the department. A discussion of the choices faculty made (and the reasons for their choices) was then facilitated.

### Participants

All participants were faculty or administrative staff at Boise State, a 4-year public institution. A total of 169 individuals completed the prompts described above. In each department meeting in which data were collected, all or nearly all, full time, and tenured/tenure-track faculty members were in attendance. In some departments, this conversation also included full-time lecturers (not on the tenure track) and department administrative staff. The intention was to engage those most responsible for driving faculty norms around teaching. While individual data sheets were completed anonymously, participants were provided an opportunity to self-identify their rank at the university. Approximately 60% of the participants elected to self-identify their rank. Of these, 85 (83.3%) were department chairs or tenured/tenure-track faculty, 11 (10.8%) were lecturers, 1 (1%) was an adjunct faculty, and 5 (4.9%) were administrative personnel. No other demographic information was collected. Each item written by participants on their response sheet was transcribed and entered into an Excel file for a total of 2792 excerpts.

### Data analysis

Participant responses to the change characteristics that referenced positive attributes of the change were considered drivers for the change, defined as a situational, physical, cultural, or personal factor (real or perceived) that aids in the progression toward the articulated vision. Likewise, responses that alluded to negative attributes of the change were considered barriers; a barrier is defined as a situational, physical, cultural, or personal factor (real or perceived) that impedes one’s ability or propensity to move toward the articulated vision. The codes used within these two large categories were developed through an inductive approach, meaning the codes were derived from the data itself rather than using pre-existing codes (Braun and Clarke 2006). In phase I, the excerpts in the first coding cycle were coded by three researchers who utilized descriptive coding to identify the basic topic of a passage; the second coding cycle utilized focused coding to develop the categories (Saldana 2016). This phase of coding resulted in the generation of 18 proposed barrier categories and 11 proposed driver categories.

In phase II, two different researchers recoded each excerpt into one of the proposed categories developed during the focused coding cycle. The two researchers collaboratively coded one department’s comments in order to clarify the meaning of each category. In that process, nuances of each category were identified and categories were either refined or new categories were identified (Saldana 2016). Coding then proceeded independently for the remaining STEM departments using 18 barrier categories and 15 driver categories. The researchers engaged in ongoing, reflexive dialog throughout the coding process (Saldana 2016; Braun and Clarke 2006)—to assure the categories were being used consistently. Intercoder agreement was initially 67.1%; the researchers then discussed each instance of disagreement on codes and attempted to reach consensus (Saldana 2016). Final analysis was characterized by an intercoder agreement of 92.5%, meaning the researchers did not reach consensus on less than 8% of items coded. In these situations, both researcher’s codes were included. The results were then counted and expressed as the percent of participants that noted a particular barrier or driver (Saldana 2016).

## Results

In this study, we sought to identify faculty reactions to a vision for teaching and learning consistent with recommendations for STEM education reform that have emerged at the national level. Tables 2 and 3 present the barrier and driver categories that emerged from the analysis of faculty comments, along with example comments that are found in each category. In each table, the categories are presented in the relative order of frequency of the category, reflected in faculty comments in the data at the aggregate level; the category with the highest percent of faculty responses is listed first. Quantitative results are presented in figures following Tables 2 and 3.

**Fig. 1:**
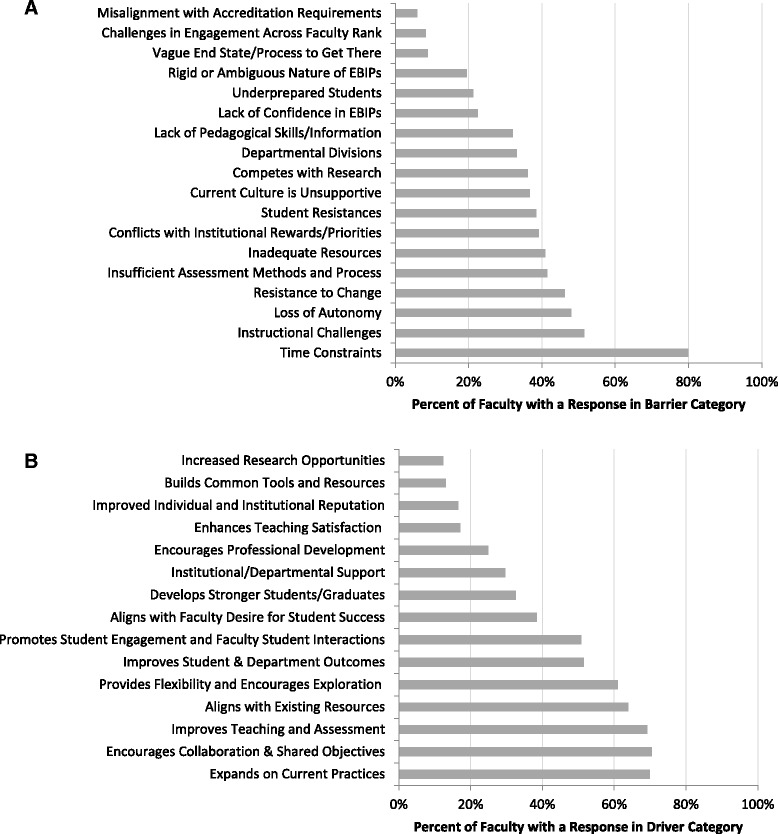
Categories of barriers (**a**) and drivers (**b**) to STEM education reform. Categories were emergent based on analysis of comments from 169 faculty, aggregated across all STEM departments. The *length of the bar* indicates the percent of participants who had a response that was coded to the respective category

Figure 1 provides a quantitative comparison of the barrier and driver category analysis aggregated for STEM departments. The most frequent barriers are “time constraints,” “instructional challenges,” “loss of autonomy,” and “resistance to change.” The data clearly indicate that “time constraints” appears much more frequently than any other barrier category. Drivers with the highest frequency across STEM departments are “expands on current practices,” “encouragement of collaboration and shared objectives,” “improves teaching and assessment,” and “aligns with existing resources.”

**Fig. 2:**
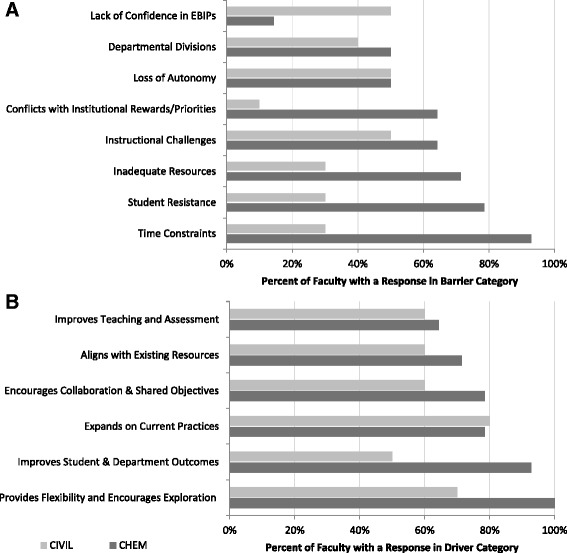
Sample department comparison: barriers (**a**) and drivers (**b**) to STEM education reform. Categories were emergent based on analysis of comments aggregated across all STEM departments. The *length of the bar* indicates the percent of participants who had a response that was coded to the respective category

Because departmental context has been shown to be important for EBIP adoption (Lund and Stains 2015), it is likely that faculty responses to the broader vision statement in this study will vary with departmental context. Therefore, it is useful to look at these results at the individual department level. A complete set of all barrier and driver data for all STEM departments is included in Additional file 1. An analysis of the departmental results reveals several similarities in the distribution frequency for each category. The “time constraints” category is contained in the top three barriers for all but one department. In addition, the most frequent driver category, “expands on current practices,” was a top driver for all but four departments. As an illustration of the variation that can exist between departments, the five highest frequency categories for the Department of Chemistry and the Department of Civil Engineering are presented in Fig. 2. For example, the Department of Chemistry’s most common barrier is “time constraints” followed by “student resistance” and “inadequate resources”. In contrast, the Department of Civil Engineering has three barriers, which appear with the same frequency: “lack of confidence in EBIPs,” “loss of autonomy,” and “instructional challenges.” Similarly, “provides flexibility and encourages exploration” is the most frequently noted driver for the Department of Chemistry, while the most frequent driver for the Department of Civil Engineering is “expands on current practices.”

## Discussion

The discussion of results is organized around the specific questions we sought to answer in this study: what barriers and drivers do faculty identify in response to a proposed shift in teaching and learning norms in STEM? How do faculty responses vary across departments? We compare our results to ideas already in the literature and discuss the implications of our results for stimulating change in STEM education.

### Barriers to a shift in teaching norms

The data presented here allow us to examine the response of faculty to a proposed shift in teaching and learning norms. In this study, faculty identified a variety of barriers; the two barriers noted most frequently in the aggregate results (Fig. 1) are “time constraints” and “instructional challenges.” These categories are similar to barriers that researchers have identified for faculty adoption of EBIPs. For example, instructors reported lack of time as a barrier to adopting active learning strategies (Henderson and Dancy 2007; Brownell and Tanner 2012). Instructional challenges documented as barriers in the literature include concerns about not being able to cover all of the course content if active learning strategies are used and other classroom management issues (Andrews and Lemons 2015; Henderson and Dancy 2007; Lund and Stains 2015; Parker et al. 2015). The instructional challenges category in the current study includes barriers such as implementation concerns related to class sizes, content coverage, meeting the diversity of student expectations, and classroom configurations. Additional barriers noted in the literature that have a parallel in the current study include the competition between research and teaching (Lester and Kezar 2012; Chasteen et al. 2015; Parker, et al. 2015), lack of institutional incentives (Walczyk et al. 2007; Chasteen, et al. 2015; Parker, et al. 2015), and a concern that students are underprepared (Felder and Brent 1996; Parker, et al. 2015) or are resistant (Henderson and Dancy 2007; Hastings and Breslow 2015; Parker, et al. 2015). The overlap between our results and those from studies focused on changes to faculty pedagogy is perhaps not surprising, given that the use of EBIPs is specifically identified as a desired component of the vision; it is clear that our faculty were responding most directly to the component of the vision calling for the exploration and adoption of EBIPs. However, several of our categories are distinct from those in previous studies. For example, the barrier of “insufficient assessment methods and processes” includes faculty responses that identify the need for clearer assessment strategies so that teaching can be “evidenced and informed by meaningful assessment.” This included both the assessment of faculty’s teaching and of student learning; specifically, how will teaching effectiveness be systematically and consistently measured across the institution for faculty and in what ways can student learning gains be documented, collected, and analyzed consistently across courses and instructors? Likewise, the identification of departmental divisions is likely a barrier related to the component of the vision calling for faculty dialog around teaching and learning. Even the “time constraints” category in our study included comments that were both about the time related to adoption of EBIPs as well as the time needed for “ongoing” efforts around teaching and learning called for in the vision. Some categories, such as (perceived) “loss of autonomy,” “resistance to change,” and “current culture is unsupportive” illuminate underlying assumptions, beliefs, or values that must be shifted if the planned change is to be successful; however, the most-frequently noted barriers tend to be pointed at logistical and structural challenges.

### Drivers for a shift in teaching norms

While understanding the barriers to change in STEM Education is important, as we seek to identify effective strategies for change, it may be equally important to identify the drivers that can be leveraged proactively to catalyze change. Faculty in the present study, responding to the proposed vision, indicated that the four most important drivers for change were that the vision “expands on current practice,” “encourages collaboration and shared objectives,” “improves teaching and assessment,” and “aligns with existing resources.”

Because less is known about drivers for change, it is valuable to unpack these most-frequently noted drivers. The category of “expands on current practice” included the following subthemes that could contribute to the momentum toward changed teaching practice: faculty could learn from their colleagues who had already adopted pedagogical or assessment practices outlined in the vision or faculty self-identified as being somewhere along the adoption curve (Rogers 2003) for changed teaching or assessment practice. The second category, “encourages collaboration and shared objectives,” appears to be largely in response to the component of the vision that calls for dialog around teaching; comments focused on the acknowledgement that having discussions within the department about teaching might be expected to result in better coordination of courses and curriculum, as well as enhanced collegiality. The comments contained in the category “improves teaching and assessment” were focused around the idea that faculty are already teaching and are, increasingly, called upon to engage in assessment; moving toward this vision would make their teaching and assessment efforts more effective. The notion that the vision “aligns with existing resources” was an acknowledgement that a move toward this vision would require some resources that were, in fact, already in place. Faculty frequently noted the resources of the university’s Center for Teaching and Learning and the accessibility and support of technology, as well as colleagues in their department or within the institution that possess pedagogical expertise.

The driver data, similar to the barrier data, identifies both structural supports (e.g., aligns with existing resources) as well as some ideas that capture underlying values that faculty hold about teaching. For example, the identification of collaboration and shared objectives as something that will drive toward the vision is reflective of the value placed on faculty working together toward a common goal. In addition, it is interesting that the top barrier and driver categories are focused on the impact such a vision would have on the faculty rather than the benefits for students. It is not until the sixth most frequently noted driver category and the eighth most frequently noted barrier category that the focal point becomes student-centered. For the drivers, comments indicate that achieving the vision would result in improved student learning and department outcomes such as increased enrollment or retention of majors. For the barriers, comments indicate student resistance to active learning pedagogies is a barrier to faculty adoption of EBIPs.

Further, similar to the barriers, the results in the present study have some resonance with those from studies that have examined factors that drive faculty adoption of EBIPs. For example, in a study of science and engineering faculty who chose to engage in professional development around teaching, faculty indicated that they were interested in increasing their teaching competencies and in interacting with others to improve their teaching (Bouwma-Gearhart 2012), consistent with the notion in our data that change would improve teaching and would provide for collaborative interactions. The interest in alignment with existing resources in our study is also consistent with work that has asserted that department level support to help with the implementation of initiatives was key for successful changes to take hold (Wieman and Gilley 2013; Hastings and Breslow 2015). For example, Wieman and Gilley (2013) investigated the rate of continued use of reformed teaching practices resulting from the Carl Wieman Science Education Initiative (CWSEI) at the University of British Columbia. They concluded that the continued use of reformed practices likely resulted from individual discipline-specific Science Education Specialists embedded in the departments and supportive department environments where the department demonstrated a commitment to transforming teaching and where faculty are engaged in ongoing dialog pertaining to their teaching efforts.

It is important to distinguish two important differences between the drivers identified in the current study and those in the literature. The driver categories in our study emerge from faculty perception of what *will* help make change occur. They are speculative rather than retrospective; they do not identify what turned out to have been helpful. Further, they emerged from feedback from faculty in all STEM departments, including faculty who are already using a variety of evidence-based pedagogical and assessment strategies, those who are interested but have not yet adopted, and those who had expressed no interest in making changes to their teaching.

### Barriers and drivers at the department level

While the above discussion about aggregate barriers and drivers is interesting and potentially useful, our results show the distribution of barriers and drivers can vary substantially from one department to another. The contrasting examples of Chemistry and Civil Engineering (Fig. 2) suggest these departments likely have different norms and are also starting from different places relative to engagement in changes to teaching practice. For example, in Chemistry, much like most other departments, time constraints are a significant barrier; however, “instructional challenges” and “inadequate resources” are also significant barriers; this particular combination of top barriers is unique among the departments in this study. In contrast, in Civil Engineering, one of the greatest barriers is “lack of confidence in EBIPs”; this is unique, as this barrier does not appear in the top three barriers for any other department. This comparison suggests that the strategies that might be employed to support shifts in teaching norms need to be tailored to departmental contexts. A department whose primary barrier is a lack of confidence in EBIPs will need discussions and support to explore the value of evidenced-based practices—something that is less necessary in a department where this is not a significant barrier. Likewise, a department that sees that moving toward the vision will “encourage flexibility and exploration” is likely to engage with different strategies than one in which a primary driver is the prospect of more graduates (“improves student and department outcomes”). If strategies can be implemented that will actually shift the local context around teaching and learning in a department (what people are doing, talking about, and valuing), there is a higher probability of movement toward the vision.

### Using barriers and drivers

The project described in this study is ongoing; faculty’s response to the vision (e.g., their perceived barriers and drivers) were collected at the start of the project and enabled the project team to work with departments to identify strategies that could be implemented to engage faculty in dialog about teaching and learning and exploration of new pedagogical and assessment practices. A detailed account of these strategies and their impact on faculty practice is not within the scope of this paper; however, Table 4 provides a few brief examples.

**Table 4:** Example strategies informed by barrier and driver categories

Barrier or driver	Example strategies
Barrier: lack of time	Mini-grants supported individual faculty or teams of faculty to explore and implement EBIPs and assessment strategies; all departments have had at least one project
Barrier: lack of pedagogical knowledge/information	A list of pedagogical strategies with discipline-specific references was created for each STEM department; one department posted this table in their lounge as a starting point for discussion.
Driver: encourages collaboration and shared objectives	In response to department activity, communities of practice were supported to engage faculty in continued exploration of specific pedagogies.
Driver: improves teaching and assessment	Data team created to assist faculty in using institutional student data to inform their teaching

## Conclusions

### Implications for Change

Gaining a better understanding of faculty-identified barriers and drivers in response to a proposed shift in the teaching and learning is incredibly valuable. As shown in the examples in Table 4, knowing the barriers and drivers allows change leaders to work with faculty to identify strategies that leverage particular drivers and work on the removal of barriers. This information can be used to increase the number of supportive individual and contextual factors present in a department (Lund and Stains 2015), which is known to impact faculty practice. For example, one way to help create a supportive environment might be to create “time” resources through course reductions or summer salary. This is an important way for an institution to both signal the importance of making changes and substantively create the space for faculty to make changes in their teaching. Strategies might further include seeding conversations about EBIPs, highlighting effective pedagogical and assessment practice already going on in a department, or facilitating communities of practice.

It is likely that not all driver or barrier categories are equally important for enacting change; the frequency with which faculty noted barriers or drivers is not necessarily directly correlated with the factors that will serve as important tipping points for change. For example, faculty most frequently note time constraints as a barrier, but it is possible that aspects of a department’s local culture, especially aspects that are supportive of teaching and learning, may be more important for actually moving transformations forward (Kezar and Holcombe 2015; Lund and Stains 2015). That said, knowing the local context well increases the chances that the strategies implemented during the change process will have an impact on shifting the teaching norms.

An important outcome of this study is the reinforcement of the notion that proposed changes will always be supported or constrained by the local context. While the categories of barriers and drivers presented here have some consistency with data presented in other studies, we cannot know if the patterns observed in this study would be reproduced at another institution. We suspect the patterns are likely a complex intersection of discipline and departmental/institutional context and history. This implies that just like a “one-size-fits-all” approach is not likely to be successful within an institution, a “one-size-fits-all” approach is unlikely to work between institutions. Thus, individual institutions should engage in data collection and analysis in order to understand their unique system first and identify the perceived barriers and drivers of *their* faculty and departments. Then, they can use their understanding to work with faculty to implement the most successful change strategies for their institution (Henderson et al. 2011).

Finally, it is important to point out that the work described herein served as an initial stage in a change process. Our project team began by considering the desired behaviors that would be observed if the institution’s teaching and learning environments were student centered. This forced us to think beyond the adoption of EBIPs and clarify the behaviors we expected to see when we achieve the long-term goal of shifting faculty conceptions about teaching and learning, the assumptions faculty make around how teaching and learning works, and what teaching looks like (Czajka and McConnell 2016; Kember 1997). As a result, we recommend institutions that desire to increase the use of EBIPs, take a more holistic approach, and propose a broader vision for the transformation of teaching, rather than focusing solely on the adoption of EBIPs. Further, asking faculty to respond to the vision was a mechanism for introducing the change, an important step in Dormant’s (2011) change model. The need to consider the faculty’s perspective, also part of Dormant’s model, led to the development of a standardized process and method for collecting faculty responses to the vision across our institution. It was important that the mechanism allowed for faculty to express their responses in terms of *both* drivers and barriers rather than just ‘receive’ the announcement of a new initiative from central administration and be expected to assume it was positive. The discussions in the meetings in which data were collected contributed to concrete interest in and activity toward changed teaching and learning practice and have served as the foundation for a change project aimed at building a shifted culture for STEM teaching at our institution (Henderson, et al. 2011).

### Limitations

Because the data were collected without also collecting information about the demographics of the individual providing the responses, we are not able to look at trends related to other variables that may be important (e.g., years of teaching or whether a faculty member had done a lot of faculty development). Also, data were collected from whomever was present at the department meeting. Because different departments have different norms about who attends meetings, the samples in the departments are not totally comparable. However, in most departments, all or nearly all of the full-time teaching faculty were present during our data gathering efforts, which gives us confidence that that the results are representative of those shaping department norms around teaching.

## Additional file

Additional file 1: Table S1. Normalized frequency of departmental comments in each barrier category. **Table S2.** Normalized frequency of departmental comments in each driver category. (DOCX 23 kb)

## Abbreviations

ABET: Accreditation Board for Engineering and Technology
ACS: American Chemical Society
EBIPs: Evidence Based Instructional Practices
NSF: National Science Foundation
STEM: Science Technology Engineering and Math
