# Supplementary material for: Faculty drivers and barriers: laying the groundwork for undergraduate STEM education reform in academic departments
Source: Int J STEM Educ. 2017 Apr 13;4(1):8. doi: 10.1186/s40594-017-0062-7 (PMC6310369; doi:10.1186/s40594-017-0062-7)
Supplement: Supplementary file 1 — Normalized frequency of departmental comments in each barrier category. Table S2. Normalized frequency of departmental comments in each driver category. (DOCX 23 kb) [file 40594_2017_62_MOESM1_ESM.docx]

**Additional file**

Table S1. Normalized Frequency of Departmental Comments in Each Barrier Category

|  | **Departments** | | | | | | | | | | | | |
| --- | --- | --- | --- | --- | --- | --- | --- | --- | --- | --- | --- | --- | --- |
| **Barriers** | Anthropology (n = 8) | Biology ( n = 17) | Chemistry (n = 15) | Civil Engineering (n = 10) | Computer Science (n = 11) | Electrical and Computer Science Engineering (n = 18) | Geosciences (n = 15) | Material Science and Engineering (n = 14) | Math (n = 23) | Mechanical and Biomedical Engineering (n = 18) | Physics (n= 12) | Psychology (n = 11) | **STEM Aggregate (n = 169)** |
| Time Constraints | *88%* | *81%* | *93%* | *30%* | *82%* | *83%* | *71%* | *93%* | *65%* | *89%* | *100%* | *82%* | ***80%*** |
| Instructional Challenges | *25%* | *75%* | *64%* | *50%* | *36%* | *50%* | *57%* | *50%* | *22%* | *50%* | *58%* | *91%* | ***51%*** |
| Loss of Autonomy | *13%* | *69%* | *50%* | *50%* | *36%* | *44%* | *36%* | *36%* | *65%* | *61%* | *67%* | *9%* | ***48%*** |
| Resistance to Change | *38%* | *44%* | *50%* | *30%* | *55%* | *39%* | *79%* | *64%* | *30%* | *39%* | *67%* | *27%* | ***46%*** |
| Insufficient Assessment Methods & Processes | *25%* | *31%* | *50%* | *30%* | *27%* | *44%* | *64%* | *50%* | *39%* | *39%* | *58%* | *27%* | ***41%*** |
| Inadequate Resources | *25%* | *13%* | *71%* | *30%* | *27%* | *17%* | *64%* | *43%* | *74%* | *28%* | *17%* | *64%* | ***41%*** |
| Conflicts with Institutional Rewards/Priorities | *50%* | *69%* | *64%* | *10%* | *0%* | *17%* | *43%* | *29%* | *35%* | *39%* | *50%* | *64%* | ***39%*** |
| Student Resistance | *13%* | *56%* | *79%* | *30%* | *9%* | *39%* | *43%* | *36%* | *22%* | *17%* | *58%* | *55%* | ***38%*** |
| Current Culture is Unsupportive | *0%* | *25%* | *43%* | *30%* | *0%* | *56%* | *43%* | *50%* | *48%* | *33%* | *58%* | *18%* | ***37%*** |
| Competes with Research | *25%* | *25%* | *50%* | *20%* | *55%* | *33%* | *29%* | *29%* | *17%* | *61%* | *58%* | *36%* | ***36%*** |
| Departmental Divisions | *13%* | *63%* | *50%* | *40%* | *9%* | *56%* | *29%* | *14%* | *17%* | *61%* | *17%* | *0%* | ***33%*** |
| Lack of Pedagogical Skills/Information | *13%* | *13%* | *36%* | *30%* | *18%* | *39%* | *29%* | *14%* | *30%* | *50%* | *67%* | *36%* | ***32%*** |
| Lack of Confidence in EBIPs | *50%* | *6%* | *14%* | *50%* | *27%* | *22%* | *21%* | *14%* | *17%* | *11%* | *50%* | *18%* | ***22%*** |
| Underprepared Students | *0%* | *25%* | *14%* | *0%* | *0%* | *22%* | *36%* | *7%* | *17%* | *17%* | *33%* | *82%* | ***21%*** |
| Rigid or Ambiguous Nature of EBIPs | *0%* | *13%* | *57%* | *20%* | *0%* | *33%* | *7%* | *7%* | *35%* | *22%* | *8%* | *0%* | ***20%*** |
| Challenges in Engagement Across Faculty Rank | *13%* | *6%* | *21%* | *0%* | *0%* | *6%* | *14%* | *0%* | *17%* | *0%* | *17%* | *0%* | ***8%*** |
| Vague end state/process to get there | *0%* | *0%* | *0%* | *10%* | *9%* | *11%* | *14%* | *14%* | *4%* | *22%* | *17%* | *0%* | ***9%*** |
| Misalignment with Accreditation Requirements | *0%* | *0%* | *43%* | *0%* | *9%* | *6%* | *0%* | *7%* | *0%* | *6%* | *0%* | *0%* | ***6%*** |

Table S2. Normalized Frequency of Departmental Comments in Each Driver Category

|  | **Departments** | | | | | | | | | | | | |
| --- | --- | --- | --- | --- | --- | --- | --- | --- | --- | --- | --- | --- | --- |
| **Drivers** | Anthropology (n = 8) | Biology ( n = 17) | Chemistry (n = 15) | Civil Engineering (n = 10) | Computer Science (n = 11) | Electrical and Computer Science Engineering (n = 18) | Geosciences (n = 15) | Mechanical and Biomedical Engineering (n = 18) | Material Science and Engineering (n = 14) | Math (n = 23) | Physics (n= 12) | Psychology (n = 11) | **STEM Aggregate (n = 169)** |
| Expands on Current Practices | *0%* | *88%* | *79%* | *80%* | *82%* | *78%* | *79%* | *79%* | *77%* | *78%* | *58%* | *18%* | ***70%*** |
| Encourages Collaboration & Shared Objectives | *25%* | *81%* | *79%* | *60%* | *55%* | *56%* | *79%* | *86%* | *77%* | *94%* | *83%* | *36%* | ***70%*** |
| Improves Teaching & Assessment | *63%* | *88%* | *64%* | *60%* | *73%* | *67%* | *86%* | *86%* | *64%* | *78%* | *83%* | *9%* | ***69%*** |
| Aligns with Existing Resources | *13%* | *63%* | *71%* | *60%* | *27%* | *61%* | *64%* | *100%* | *68%* | *72%* | *50%* | *91%* | ***64%*** |
| Provides Flexibility & Encourages Exploration | *25%* | *69%* | *100%* | *70%* | *45%* | *61%* | *71%* | *64%* | *45%* | *56%* | *75%* | *45%* | ***61%*** |
| Improves Student & Department Outcomes | *75%* | *69%* | *93%* | *50%* | *55%* | *61%* | *43%* | *50%* | *23%* | *50%* | *67%* | *0%* | ***51%*** |
| Promotes Student Engagement & Faculty Student Interactions | *25%* | *69%* | *50%* | *50%* | *27%* | *56%* | *50%* | *71%* | *64%* | *44%* | *67%* | *9%* | ***51%*** |
| Aligns with Faculty Desire for Student Success | *75%* | *69%* | *57%* | *40%* | *18%* | *0%* | *43%* | *43%* | *36%* | *33%* | *33%* | *36%* | ***38%*** |
| Develops Stronger Students/Graduates | *38%* | *38%* | *21%* | *40%* | *45%* | *39%* | *57%* | *29%* | *32%* | *33%* | *8%* | *9%* | ***33%*** |
| Institutional/Departmental Support | *38%* | *25%* | *29%* | *20%* | *36%* | *22%* | *21%* | *36%* | *18%* | *39%* | *33%* | *55%* | ***30%*** |
| Encourages Professional Development | *25%* | *25%* | *36%* | *20%* | *9%* | *22%* | *29%* | *29%* | *14%* | *28%* | *17%* | *55%* | ***25%*** |
| Enhances Teaching Satisfaction | *0%* | *19%* | *21%* | *10%* | *9%* | *22%* | *21%* | *0%* | *36%* | *33%* | *0%* | *0%* | ***17%*** |
| Improved Individual & Institutional Reputation | *13%* | *19%* | *21%* | *10%* | *27%* | *6%* | *29%* | *29%* | *23%* | *6%* | *17%* | *0%* | ***17%*** |
| Builds Common Tools & Resources | *0%* | *38%* | *43%* | *0%* | *9%* | *6%* | *0%* | *0%* | *5%* | *28%* | *0%* | *18%* | ***13%*** |
| Increased Research Opportunities | *0%* | *19%* | *14%* | *20%* | *9%* | *0%* | *29%* | *14%* | *18%* | *6%* | *8%* | *9%* | ***12%*** |
